# PD-1单抗联合化疗与化疗治疗III-IV期*SMARCA4*缺失型非小细胞肺癌的疗效评估及预后分析

**DOI:** 10.3779/j.issn.1009-3419.2023.101.26

**Published:** 2023-09-20

**Authors:** Xinjuan WANG, Meng TU, Hongxia JIA, Hongping LIU, Yan WANG, Yibo WANG, Nan JIANG, Chunya LU, Guojun ZHANG

**Affiliations:** 450000 郑州，郑州大学第一附属医院呼吸内科; Department of Pulmonary Medicine, The First Affiliated Hospital of Zhengzhou University, Zhengzhou 450000, China

**Keywords:** 肺肿瘤, SMARCA4, 免疫检查点抑制剂, 化疗, 预后, Cox比例风险模型, Lung neoplasms, SMARCA4, Immune checkpoint inhibitors, Chemotherapy, Prognosis, Cox proportional hazards models

## Abstract

**背景与目的** SMARCA4突变广泛分布于各种人类肿瘤中，具有SMARCA4突变的非小细胞肺癌（non-small cell lung cancer, NSCLC）至少占所有NSCLC的10%。由于SMARCA4缺失型非小细胞肺癌（SMARCA4-deficient non-small cell lung cancer, SMARCA4-DNSCLC）具有高侵袭性和难治性，且缺乏靶向药物治疗的敏感位点突变，常规放疗和靶向治疗难以改善患者预后，联合或不联合免疫治疗仍是其主要治疗方案，但对于SMARCA4-DNSCLC的有效治疗方案仍存在争议。本研究旨在探讨程序性死亡受体1（programmed cell death 1, PD-1）单抗联合化疗与化疗在III-IV期SMARCA4-DNSCLC患者中的疗效及预后。**方法** 回顾性分析46例III-IV期SMARCA4-DNSCLC患者的一般资料，并探讨其预后的影响因素，按治疗方案将其分为PD-1单抗联合化疗组及化疗组，对两组患者进行疗效评估和生存分析。**结果** SMARCA4-DSCLC多发生于男性吸烟患者。PD-1单抗联合化疗组与化疗组间的客观缓解率（76.5% vs 69.0%, P=0.836）及疾病控制率（100.0% vs 89.7%, P=0.286）差异无统计学意义；PD-1单抗联合化疗组1年生存率为62.7%，化疗组为46.0%；PD-1单抗联合化疗组与化疗组中位无进展生存期（progression-free survival, PFS）差异具有统计学意义（9.3个月 vs 6.1个月，P=0.048）。Cox回归分析结果显示治疗方案及吸烟史是III-IV期SMARCA4-DNSCLC患者PFS的独立影响因素，家族史是III-IV期SMARCA4-DNSCLC患者总生存期的独立影响因素。**结论** 治疗方案是III-IV期SMARCA4-DNSCLC患者预后的影响因素，PD-1单抗联合化疗患者预后可能更好。

## Abstract (en)

**Background and objective** The SMARCA4 mutation has been shown to account for at least 10% of non-small cell lung cancer (NSCLC). In the present, conventional radiotherapy and targeted therapy are difficult to improve outcomes due to the highly aggressive and refractory nature of SMARCA4-deficient NSCLC (SMARCA4-DNSCLC) and the absence of sensitive site mutations for targeted drug therapy, and chemotherapy combined with or without immunotherapy is the main treatment. Effective SMARCA4-DNSCLC therapeutic options, however, are still debatable. Our study aimed to investigate the efficacy and prognosis of programmed cell death 1 (PD-1) immune checkpoint inhibitors (ICIs) in combination with chemotherapy and chemotherapy in patients with stage III-IV SMARCA4-DNSCLC. **Methods ** 46 patients with stage III-IV SMARCA4-DNSCLC were divided into two groups based on their treatment regimen: the chemotherapy group and the PD-1 ICIs plus chemotherapy group, and their clinical data were retrospectively analyzed. Efficacy assessment and survival analysis were performed in both groups, and the influencing factors for prognosis were explored for patients with SMARCA4-DNSCLC. **Results** Male smokers are more likely to develop SMARCA4-DNSCLC. There was no significant difference in the objective response rate (76.5% vs 69.0%, P=0.836) between chemotherapy and the PD-1 ICIs plus chemotherapy or the disease control rate (100.0% vs 89.7%, P=0.286). The one-year overall survival rate in the group with PD-1 ICIs plus chemotherapy was 62.7%, and that of the chemotherapy group was 46.0%. The difference in median progression-free survival (PFS) between the PD-1 ICIs plus chemotherapy group and the chemotherapy group was statistically significant (9.3 mon vs 6.1 mon, P=0.048). The results of Cox regression analysis showed that treatment regimen and smoking history were independent influencing factors of PFS in patients with stage III-IV SMARCA4-DNSCLC, and family history was an individual influencing factor of overall survival in patients with stage III-IV SMARCA4-DNSCLC. **Conclusion ** Treatment regimen may be a prognostic factor for patients with SMARCA4-DNSCLC, and patients with PD-1 ICIs plus chemotherapy may have a better prognosis.

SMARCA4是位于染色体19p13.2上编码SMARCA4/BRG1蛋白的抑癌基因^[1]^，该蛋白是SWI/SNF复合物的重要组成亚基之一，SMARCA4突变可能引起SMARCA4/BRG1蛋白表达缺失。SWI/SNF复合物是一种在进化上高度保守的ATP依赖性染色质重塑复合物，是最早被发现和熟知的染色质重塑复合物，在重要的细胞进程和功能调控，如基因的转录、增殖、分化和DNA修复等方面发挥重要作用^[2]^，研究^[3]^表明SWI/SNF复合体具有重要的抑癌作用，它由近22个基因编码的至少15个蛋白质亚基组装而成，但是发挥核心作用的是依赖ATP酶的催化亚基，包括SMARCA2和SMARCA4。在所有恶性肿瘤中5%-7%的SMARCA4发生突变，包括肺癌、结肠癌、膀胱癌、乳腺癌、卵巢高钙血症型小细胞癌、胸部肉瘤和子宫肉瘤^[4⇓-6]^。在肺癌中，催化亚基SMARCA4的失活是SWI/SNF复合物中最常见的改变，约10%的NSCLC中存在SMARCA4突变^[7]^，这与患者的不良预后相关^[3,8,9]^。SMARCA4缺失型非小细胞肺癌（SMARCA4-deficient non-small cell lung cancer, SMARCA4-DNSCLC）具有较高的侵袭性和难治性，缺乏靶向药物治疗的敏感位点突变，传统放化疗和靶向治疗难以改善患者生存。随着免疫治疗的不断发展，免疫检查点抑制剂（immune checkpoint inhibitors, ICIs）在NSCLC患者的疗效及预后改善方面发挥了重大作用，但在SMARCA4-DNSCLC中的作用尚缺少相关研究。本研究旨在探讨程序性死亡受体1（programmed cell death 1, PD-1）单抗联合化疗与化疗在III-IV期SMARCA4-DNSCLC患者中的疗效及预后。

## 1 资料与方法

### 1.1 一般资料

回顾性收集2020年1月1日至2022年12月31日于我院确诊为SMARCA4-DNSCLC患者的临床资料。纳入标准：（1）年龄≥18岁；（2）组织病理确诊为SMARCA4-DNSCLC；（3）临床分期为III-IV期；（4）于我院规律治疗至少2个周期。排除标准：（1）临床资料不完整，疗效评价信息缺失；（2）合并其他部位原发肿瘤病史；（3）合并免疫性疾病；（4）联合手术治疗。最终共纳入46例患者。本研究得到郑州大学第一附属医院伦理委员会批准（No.2021-KY-0302）。

### 1.2 资料收集

从电子病例系统中收集患者的一般临床资料，包括性别、年龄、吸烟史、肺癌家族史、治疗方案、东部合作肿瘤小组体能状态（Eastern Cooperative Oncology Group performance status, ECOG PS）评分等。收集检验信息包括程序性死亡配体1（programmed death ligand 1, PD-L1）表达状态、治疗前血常规、血生化、血凝、肿瘤标志物、Ki-67、肿瘤原发灶-淋巴结-转移（tumor-node-metastasis, TNM）分期、远处转移部位、基因检测结果（主要包括TP53、KRAS、PI3KCA、STK11）等。

### 1.3 疗效评估及随访

通过电子病历或电话咨询方式进行随访，末次随访时间为2023年2月1日。通过患者的影像学检查[胸部计算机断层扫描（computed tomography, CT）、腹部CT、头颅核磁共振（magnetic resonance imaging, MRI）、全身骨扫描、正电子发射断层显像计算机体层摄影术（positron emission tomography/CT, PET/CT）、彩超等]进行疗效评价。疗效评价采用实体瘤疗效评估标准1.1版，分为完全缓解（complete response, CR）、部分缓解（partial response, PR）、疾病稳定（stable disease, SD）和疾病进展（progressive disease, PD）。疾病控制率（disease control rate, DCR）=（CR+PR+SD）例数/总例数×100%。客观缓解率（objective response rate, ORR）=（CR+PR）例数/总例数×100%。无进展生存期（progression-free survival, PFS）定义为接受治疗开始到观察到疾病进展或者因任何原因死亡的时间。总生存期（overall survival, OS）定义为接受治疗开始到因任何原因死亡的时间。本研究主要观察终点为PFS、OS。

### 1.4 统计学方法

应用SPSS 26.0、GraphPad Prism 9.5.1进行分析与绘图。符合正态分布的计量资料以均数±标准差表示，采用t检验。不符合正态分布的计量资料以中位数和范围表示，采用Mann-Whitney U检验。计数资料以例数或百分比表示，采用卡方检验进行比较；应用Kaplan-Meier法进行生存分析，Log-rank检验进行组间比较。使用Cox比例风险模型进行单因素及多因素分析。P<0.05为差异有统计学意义，P值均为双侧检验。

## 2 结果

### 2.1 患者临床特征

共纳入SMARCA4-DNSCLC患者46例。65岁及以下的占54.3%（25/46）；男性占97.8%（45/46）；有吸烟史的占73.9%（34/46）；中心型肺癌占58.7%（27/46）；ECOG PS评分为0-1分的占60.9%（28/46）；IV期患者占58.7%（27/46）。根据治疗方案将患者分为化疗组29例（63.0%）和PD-1单抗联合化疗组17例（37.0%）；44例患者选用含铂方案化疗，根据铂类不同分为顺铂组6例（13.0%）和非顺铂组38例（82.6%）。经治后，无患者达到CR，71.7%（33/46）的患者达到PR，21.7%（10/46）达到SD，6.6%（3/46）达到PD。截止到观察终点，3例患者失访，37.0%（17/46）的患者观察到死亡，56.5%（26/46）存活，46例入组患者的基线资料及PD-1单抗联合化疗与化疗组基线比较见表1。

**表1:** 46例SMARCA4-DNSCLC患者的临床特征及PD-1单抗联合化疗组与化疗组患者基线资料对比

Variable	All patients (n=46)	Chemotherapy (n=29)	PD-1 ICIs plus chemotherapy (n=17)	P
Age (yr), Mean±SD	63.00±7.93	63.14±7.23	60.29±8.94	0.145
Male, n (%)	45 (97.8)	28 (96.6)	17 (100.0)	0.630
ECOG PS, 0-1, n (%)	28 (60.9)	17 (58.6)	11 (64.7)	0.683
Smoking, n (%)	34 (73.9)	22 (75.9)	12 (70.6)	0.964
Family history, n (%)	6 (13.0)	6 (20.7)	0 (0.0)	0.051
T stage, 1+2, n (%)	27 (58.7)	18 (62.1)	9 (52.9)	0.544
Location, Central, n (%)	27 (58.7)	17 (58.6)	10 (58.8)	0.989
Brain metastasis, n (%)	11 (23.9)	6 (21.4)	5 (31.3)	0.717
Bone metastasis, n (%)	14 (30.4)	9 (31.0)	5 (29.4)	0.848
Adrenal metastasis, n (%)	6 (13.0)	4 (13.8)	2 (11.8)	0.611
TNM, n (%)				0.989
III	19 (41.3)	12 (41.4)	7 (41.2)	
IV	27 (58.7)	17 (58.6)	10 (58.8)	
PD-L1, n (%)				0.579
Negative	16 (34.8)	10 (34.5)	6 (35.3)	
Positive	17 (37.0)	9 (31.0)	8 (47.1)	
Unknown	13 (28.2)	10 (34.5)	3 (17.7)	
Erythrocytes (×10^12^/L), Median (P_25_, P_75_)	7.66 (6.09, 8.90)	7.69 (6.50, 8.93)	7.59 (5.91, 8.76)	0.918
Prothrombin time (s), Median (P_25_, P_75_)	11.30 (10.60, 12.10)	11.30 (10.60, 12.20)	11.30 (10.65, 11.95)	0.855
D-Dimer (mg/L), Median (P_25_, P_75_)	0.38 (0.16, 0.78)	0.38 (0.16, 0.80)	0.45 (0.21, 0.84)	0.657
LDH>245 U/L, n (%)	17 (37.0)	11 (37.9)	6 (35.3)	0.858
CA724>6.9 U/mL, n (%)	19 (41.3)	10 (34.5)	9 (52.9)	0.220
CA211>3.3 ng/mL, n (%)	17 (36.0)	11 (37.9)	6 (35.3)	0.858
CEA>5 ng/mL, n (%)	23 (50.0)	15 (51.7)	8 (47.1)	0.760
Ki-67>30%, n (%)	32 (69.5)	21 (72.4)	11 (64.7)	0.583
Cisplatin, n (%)	6 (13.0)	5 (17.9)	1 (6.3)	0.276
Efficacy evaluation, n (%)				
CR	0 (0.0)	0 (0.0)	0 (0.0)	
PR	33 (71.7)	20 (69.0)	13 (76.5)	0.836
ORR (PR+CR)	33 (71.7)	20 (69.0)	13 (76.5)	0.836
SD	10 (21.7)	6 (20.7)	4 (23.5)	>0.999
DCR (PR+CR+SD)	43 (93.4)	26 (89.7)	17 (100.0)	0.286
PD	3 (6.6)	3 (10.3)	0 (0.0)	0.286
Death, n (%)	17 (37.0)	13 (44.8)	4 (23.5)	0.133

SMARCA4-DNSCLC: SMARCA4-deficient non-small cell lung cancer; ECOG PS: Eastern Cooperative Oncology Group performance status; PD-1: programmed cell death 1; PD-L1: programmed cell death ligand 1; ICIs: immune checkpoint inhibitors; LDH: lactic dehydrogenase; CA: carbohydrate antigen; CEA: carcinoembryonic antigen; CR: complete response; PR: partial response; SD: stable disease; PD: progressive disease; ORR: objective response rate; DCR: disease control rate; TNM: tumor-node-metastasis.

### 2.2 患者基因检测结果

对46例SMARCA4-DNSCLC患者的基因检测结果，主要包括TP53、KRAS、PI3KCA、STK11进行统计；共11例患者检测TP53基因，10例发生突变，8例为错义突变，2例为无义突变；33例患者检测KRAS基因，5例发生突变，其中4例均为2号外显子错义突变；19例患者检测PI3KCA基因，均未突变；9例患者检测STK11基因，2例发生突变，1例为移码突变，1例为错义突变；34例患者检测ALK基因，均未突变；34例患者检测EGFR基因，1例发生拷贝数扩增；34例患者检测ROS1基因，2例发生错义突变（表2）。

**表2:** 46例SMARCA4-DNSCLC患者的基因检测结果

Gene status	TP53 (n=46)	KRAS (n=46)	PI3KCA (n=46)	STK11 (n=46)	ALK (n=46)	EGFR (n=46)	ROS1 (n=46)
Mutation	10	5	0	2	0	1	2
No-mutation	1	28	19	7	34	33	32
Unknown	35	13	27	37	12	12	11

EGFR: epidermal growth factor receptor; ALK: anaplastic lymphoma kinase; ROS1: ROS proto-oncogene 1-receptor.

### 2.3 不良反应

 46例患者中，共有89.1%（41/46）的患者发生了不同程度的不良反应（表3），其中最常见的不良反应为贫血（65.2%）、恶心（65.2%）和食欲减退（52.2%）。在PD-1单抗联合化疗组，5.9%（1/17）的患者在接受了2次治疗后出现了严重的皮疹，导致更换治疗方案；共8.7%（4/46）的患者出现甲状腺功能减退，其中化疗组出现1例，PD-1单抗联合化疗组出现3例，但均未导致更换治疗方案。

**表3:** 46例SMARCA4-DNSCLC患者的不良反应

Adverse events	All patients (n=46)	Chemotherapy (n=29)	PD-1 ICIs plus chemotherapy (n=17)
Leukopenia, n (%)	10 (21.7)	7 (24.1)	3 (17.6)
Anemia, n (%)	30 (65.2)	17 (58.6)	13 (76.5)
Thrombocytopenia, n (%)	10 (21.7)	7 (24.1)	3 (17.6)
Nausea, n (%)	30 (65.2)	18 (62.1)	12 (70.6)
Vomiting, n (%)	14 (30.4)	9 (31.0)	5 (29.4)
Decreased appetite, n (%)	24 (52.2)	17 (58.6)	7 (41.2)
Deglutition disorders, n (%)	6 (13.0)	5 (17.2)	1 (5.9)
Exanthema, n (%)	1 (2.2)	0 (0)	1 (5.9)
Thyroid function abnormality, n (%)	4 (8.7)	1 (3.4)	3 (17.6)

### 2.4 PD-1单抗联合化疗组与化疗组的疗效评估

#### 2.4.1 46例SMARCA4-DNSCLC患者的疗效比较

在46例SMARCA4-DNSCLC患者中，总体ORR达71.7%，DCR达93.4%。在PD-1单抗联合化疗组，76.5%（13/17）的患者达到PR，23.5%（4/17）达到SD，ORR达76.5%，DCR达100.0%；在化疗组，69.0%（20/29）的患者达到PR，20.7%（6/29）达到SD，10.3%（3/29）达到PD，ORR达69.0%，DCR达89.7%。但两组的ORR（69.0% vs 76.5%, P=0.836）及DCR（89.7% vs 100%, P=0.286）差异无统计学意义（表1）。

#### 2.4.2 生存分析比较

在全组46例SMARCA4-DNSCLC患者中，OS的中位随访时间为10.3个月（范围：2.2-30.9个月），PFS的中位随访时间为7.3个月（范围：1.4-30.9个月）。OS的Kaplan-Meier生存分析结果显示，全组46例SMARCA4-DNSCLC患者的中位OS为12.5个月，化疗组中位OS为12个月，PD-1单抗联合化疗组中位OS未到达；PD-1单抗联合化疗组1年OS率为62.7%，化疗组1年OS率为46.0%，差异无统计学意义（P=0.237，图1A）。PFS的Kaplan-Meier生存分析结果显示PD-1单抗联合化疗组中位PFS为9.3个月（范围：1.8-30.9个月），化疗组中位PFS为6.1个月（范围：1.4-13.9个月），差异有统计学意义（P=0.048）（图2A）。顺铂组中位PFS为8.0个月，其他铂类组中位PFS为6.6个月，差异无统计学意义（P=0.959，表5）。

**图1:**
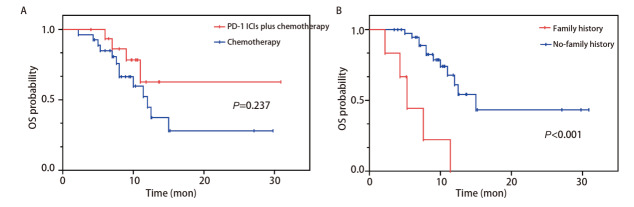
OS的生存曲线。A：PD-1单抗联合化疗组与化疗组的生存曲线；B：有家族史组与无家族史组患者的生存曲线。

**图2:**
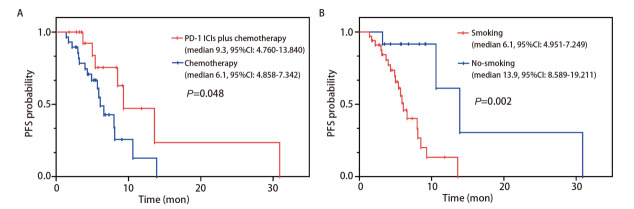
PFS的生存曲线。A：PD-1单抗联合化疗组与化疗组的生存曲线；B：有吸烟史组与无吸烟史组患者的生存曲线。

### 2.5 预后影响因素分析

#### 2.5.1 对于43例患者OS的Cox回归分析

关于OS的单因素Cox比例风险模型分析得出，吸烟史、家族史、肾上腺转移显示出统计学差异（P<0.05），而治疗方案与OS无关（P>0.05）（表4），但考虑到治疗方案具有重要的临床意义，也将其纳入多因素Cox回归分析中，结果显示癌症家族史是影响SMARCA4-DNSCLC患者OS的独立影响因素（HR=0.215, 95%CI: 0.055-0.839, P=0.027）（表4），无家族史患者预后较好（P<0.001）（图1B）。

**表4:** 46例SMARCA4-DNSCLC患者OS单因素与多因素Cox回归分析

Characteristics	Univariate analysis			Multivariate analysis	
	HR (95%CI)	P		HR (95%CI)	P
Age (<65 yr vs ≥65 yr)	0.538 (0.203-1.431)	0.214			
ECOG PS (0-1 vs 2)	0.888 (0.334-2.356)	0.811			
Smoking (Yes vs No)	0.102 (0.013-0.789)	0.029		0.131 (0.016-1.078)	0.059
Family history (Yes vs No)	0.130 (0.042-0.359)	<0.001		0.215 (0.055-0.839)	0.027
Location (Central vs Peripheral)	1.496 (0.548-4.087)	0.432			
TNM stage (III vs IV)	0.576 (0.201-1.646)	0.303			
T stage (1-2 vs 3-4)	1.067 (0.404-2.817)	0.896			
Brain metastasis (Yes vs No)	0.396 (0.149-1.050)	0.063			
Bone metastasis (Yes vs No)	1.461 (0.509-4.189)	0.481			
Adrenal metastasis (Yes vs No)	0.323 (0.112-0.934)	0.037		0.969 (0.272-3.450)	0.962
Erythrocytes	0.409 (0.153-1.093)	0.075			
Prothrombin time	0.983 (0.780-1.241)	0.888			
D-Dimer	1.306 (0.684-2.494)	0.419			
LDH (>245 U/L vs ≤245 U/L)	0.923 (0.339-2.513)	0.876			
CA724 (>6.9 ng/mL vs ≤6.9 ng/mL)	0.765 (0.293-1.999)	0.585			
CA211 (>3.3 ng/mL vs ≤3.3 ng/mL)	0.838 (0.308-2.275)	0.728			
CEA (>5 ng/mL vs ≤5 ng/mL)	1.127 (0.433-2.937)	0.808			
Ki-67 (>30% vs ≤30%)	0.657 (0.228-1.895)	0.437			
Treatment (Chemotherapy vs PD-1 ICIs plus chemotherapy)	1.945 (0.630-6.001)	0.247		0.757 (0.224-2.554)	0.653
Cisplatin (Yes vs No)	0.407 (0.127-1.303)	0.130			

#### 2.5.2 对于46例患者PFS的Cox回归分析

关于PFS的单因素Cox比例风险模型分析发现，治疗方案、凝血酶原时间、吸烟史、家族史、肾上腺转移与较长的PFS相关（P<0.05）；将其纳入多因素Cox回归分析发现，治疗方案和吸烟史是影响SMARCA4-DNSCLC患者PFS的独立影响因素（P<0.05），化疗出现PD的风险相对较高（HR=4.275, 95%CI: 1.238-14.756, P=0.022），非吸烟患者的PD风险低于吸烟患者（HR=0.110, 95%CI: 0.020-0.589, P=0.010）（表5），无吸烟史患者中位PFS更长（13.9个月 vs 6.1个月，P=0.002）（图2B）。

**表5:** 46例SMARCA4-DNSCLC患者PFS单因素与多因素Cox回归分析

Characteristics	Univariate analysis			Multivariate analysis	
	HR (95%CI)	P		HR (95%CI)	P
Age (<65 yr vs ≥65 yr)	0.508 (0.205-1.261)	0.144			
ECOG PS (0-1 vs 2)	1.068 (0.436-2.618)	0.885			
Smoking (Yes vs No)	0.139 (0.032-0.603)	0.008		0.110 (0.020-0.589)	0.010
Family history (Yes vs No)	0.293 (0.093-0.921)	0.036		0.569 (0.165-1.965)	0.373
Location (Central vs Peripheral)	1.518 (0.604-3.815)	0.374			
TNM stage (III vs IV)	0.829 (0.337-2.041)	0.683			
T stage (1-2 vs 3-4)	1.390 (0.553-3.493)	0.484			
Brain metastasis (Yes vs No)	0.525 (0.220-1.255)	0.147			
Bone metastasis (Yes vs No)	1.971 (0.712-5.452)	0.191			
Adrenal metastasis (Yes vs No)	0.342 (0.123-0.953)	0.040		0.419 (0.146-1.203)	0.106
Erythrocytes	0.531 (0.218-1.293)	0.163			
Prothrombin time	1.118 (1.013-1.234)	0.027		1.092 (0.978-1.220)	0.118
D-Dimer	1.252 (0.838-1.871)	0.272			
LDH (>245 U/L vs ≤245 U/L)	0.670 (0.293-1.534)	0.343			
CA724 (>6.9 ng/mL vs ≤6.9 ng/mL)	0.526 (0.229-1.208)	0.130			
CA211 (>3.3 ng/mL vs ≤3.3 ng/mL)	0.698 (0.304-1.603)	0.397			
CEA (>5 ng/mL vs ≤5 ng/mL)	0.548 (0.229-1.312)	0.177			
Ki-67 (>30% vs ≤30%)	0.500 (0.190-1.311)	0.158			
Treatment (Chemotherapy vs PD-1 ICIs plus chemotherapy)	2.605 (1.007-6.742)	0.048		4.275 (1.238-14.756)	0.022
Cisplatin (Yes vs No)	1.033 (0.300-3.557)	0.959			

## 3 讨论

根据2020年全球癌症统计数据^[10]^显示，肺癌占全球新发恶性肿瘤的11.4%，是第二常见恶性肿瘤，但肺癌仍是癌症死亡的首要原因，占所有癌症死亡的18.0%。虽然有很多方法用于肺癌患者的治疗，但5年生存率仍较低。研究^[11]^表明，除了与致癌因素相关外，肺癌的发生还与非致癌因素或抑癌基因突变有关。Schoenfeld等^[7]^研究表明约10%的NSCLC中存在抑癌基因SMARCA4突变，SMARCA4-DNSCLC预后较差，并首次提出这是一类具有独特分子遗传学、形态学及免疫表型特征的罕见NSCLC。SMARCA4-DNSCLC多发于中老年男性吸烟患者，这与我们的研究结果相符。研究^[11]^发现SMARCA4-DNSCLC患者中位生存时间为15.6个月，Reisman等^[8]^发现6例SMARCA4阴性表达的NSCLC患者1年生存率为33%，2年生存率为17%，3年生存率为0%，研究^[12,13]^均表明，SMARCA4-DNSCLC患者的预后差。我们的研究提示SMARCA4-DNSCLC患者中位生存时间为12.5个月，化疗组中位生存时间为12.0个月，预后更差，这可能也与我们研究纳入的患者均为III-IV期相关。在1年OS率上，PD-1单抗联合化疗组优于化疗组（62.7% vs 46.0%）。这都进一步说明了SMARCA4-DNSCLC患者预后不佳。此外，我们的研究发现，SMARCA4基因可能更常与TP53发生共突变，而极少与表皮生长因子受体（epidermal growth factor receptor, EGFR）、间变性淋巴瘤激酶（anaplastic lymphoma kinase, ALK）、肉瘤致癌因子受体（ROS proto-oncogene 1-receptor, ROS1）等可使用靶向药物治疗的位点共同突变^[7,14]^，因此化疗及免疫治疗仍是其主要治疗方法，但目前尚无明确有效的治疗方案。

作为经典的抗肿瘤治疗方案，含铂化疗是SMARCA4-DNSCLC患者的首选治疗方法，但其疗效仍存在争议。Park等^[15]^的研究表明SWI/SNF复合物参与DNA损伤的修复，而含铂的化疗方案，可诱导DNA损伤导致肿瘤细胞凋亡^[16]^，SWI/SNF染色质重塑复合物的稳定敲低可增强肿瘤细胞对顺铂的敏感性^[17]^，所以在理论上，作为SWI/SNF的核心催化亚基之一，SMARCA4缺失的肿瘤应该对此类基因毒性药物敏感。Kothandapani等^[17]^的研究显示稳定敲除BRG1的细胞DNA双链断裂修复能力降低，对顺铂的敏感性增加。有研究^[18]^整合了两项试验数据发现，与中、高表达组相比，SMARCA4低表达组的OS较短，接受顺铂联合长春瑞滨方案治疗的SMARCA4低表达患者5年疾病特异性生存期（disease-specific survival, DSS）较高^[19]^，最终得出结论，SMARCA4的低表达可增加NSCLC对铂基化疗的敏感性^[12]^。相反地，SMARCA4缺失与NSCLC患者的化疗耐药性相关，SMARCA4缺失在化疗诱导的细胞凋亡过程中产生抑制作用^[20]^。在本研究中，顺铂化疗的患者6例，其他铂类化疗的38例，我们发现顺铂化疗患者的中位PFS与其他铂类相比，差异无统计学意义（8.0个月 vs 6.6个月，P=0.959），可能与本研究中纳入病例数较少、选择顺铂化疗方案的个案数更少相关，这需要大型前瞻性研究进一步探讨顺铂在SMARCA4-DNSCLC患者中的作用。

近年来，随着免疫治疗的发展，ICIs在肺癌中的应用也取得了巨大进展，但在SMARCA4-DNSCLC患者中的疗效仍待商榷。有研究^[21]^表明，免疫治疗与SMARCA4缺失肿瘤患者的预后改善相关。一项个案报道了1例43岁的SMARCA4-DNSCLC青年男性患者，行左上叶肺切除术后2个月出现多发肺内转移，PD-L1阴性表达，四线选择纳武利尤单抗免疫治疗，肺转移病灶明显减少达14个月以上^[22]^，说明SMARCA4-DNSCLC可能对免疫治疗出现持久反应。但大多研究^[23,24]^表明SMARCA4-DNSCLC患者并不能从免疫治疗中获益，SMARCA4突变是导致肺腺癌非免疫治疗或免疫治疗不良临床结局的遗传因素，特别是KRAS突变的SMARCA4-DNSCLC患者免疫治疗结果更差。由于样本量不足，我们并未对SMARCA4-DNSCLC患者进行KRAS突变相关亚组分析，这需要在未来的研究中进一步阐述。

我们的研究发现，与单纯接受化疗相比，SMARCA4-DNSCLC患者从PD-1单抗联合化疗方案中获益更多。研究^[25,26]^表明，化疗除了可以直接杀伤肿瘤细胞外，还可以正向调节机体抗肿瘤的免疫应答，增加肿瘤细胞对ICIs的敏感性；而ICIs下调细胞内PD-L1表达又可以增加患者对铂基化疗的敏感性。Chu等^[25]^的研究验证了这点，他们通过对46个试验的3160例NSCLC患者进行meta分析，最终发现化疗联合免疫治疗组的PFS较化疗组显著延长，差异具有统计学意义（HR=0.48, 95%CI: 0.41-0.56）。同样地，我们的研究发现在SMARCA4-DNSCLC中，PD-1单抗联合化疗患者与化疗患者的中位PFS差异具有统计学意义（9.3个月 vs 6.个月，P<0.05），提示PD-1单抗联合化疗可能有效改善SMARCA4-DNSCLC预后，这对SMARCA4-DNSCLC患者的治疗具有一定的指导意义。

另外，我们通过Cox多因素回归分析发现吸烟史是SMARCA4-DNSCLC患者PFS的独立影响因素，通过绘制Kaplan-Meier生存曲线可以看出，非吸烟患者与吸烟患者的PFS差异具有统计学意义（P=0.002），提示在SMARCA4-DNSCLC中吸烟患者预后可能较差。同样地，在吸烟对肺癌影响的研究中发现，从不吸烟患者的生存率更高^[27]^。这可能与烟草中含有致癌物质，如苯并芘、尼古丁、亚硝胺等有关。Kalra等^[28]^通过动物实验得出，长期吸烟或暴露于香烟烟雾，可导致T细胞无能，使机体抗肿瘤免疫能力下降。与不吸烟患者相比，吸烟患者免疫微环境中CD8^+ ^T细胞、活化CD4^+ ^T细胞和M1巨噬细胞比例较高^[29]^，PD-L1表达更高^[30]^，这些都提示吸烟患者较非吸烟患者从ICIs中获益的潜在性更高。在一项NSCLC与免疫治疗相关的大型回顾性研究^[31]^中发现，与从不吸烟者和轻度吸烟者相比，重度吸烟者有更长的中位PFS和中位缓解持续时间（duration of response, DOR），也验证了上述观点。由于样本量不足，我们未在行免疫治疗的SMARCA4-DNSCLC患者中进行亚组分析验证这一结论。

在通过Cox多因素回归分析对SMARCA4-DNSCLC患者的OS进行研究时，与既往研究^[32,33]^结果相一致，我们发现肺癌家族史是其独立影响因素，这可能与其生活方式及饮食习惯相关，这些习惯可能在家庭中传承。但也有相反的结论，Drake等^[34]^的研究表明，有癌症家族史的患者更积极寻求与健康相关的行为改变，包括定期体育活动和戒烟/戒酒，从而改善生存结果，获得更好的预后。

两组的总体安全性相似，最常见的不良反应主要包括恶心、食欲减退和贫血，这与既往研究^[35]^相符。我们仅在PD-1单抗联合化疗组患者中发现了1例导致停药的不良反应，患者在应用2个周期免疫治疗后出现严重的皮疹，考虑与免疫治疗相关可能性大，予以停药并激素治疗后病情恢复，后续停用免疫治疗。因本研究样本量有限，我们仅对患者的不良反应进行了描述，并未进一步进行差异性分析。

本研究回顾性分析了46例III-IV期SMARCA4-DNSCLC患者，Cox比例风险模型发现治疗方案及吸烟史是SMARCA4-DNSCLC患者PFS的独立影响因素，PD-1单抗联合化疗可能改善这些患者的PFS；肺癌家族史为其OS的独立影响因素，但治疗方案与OS无关，这提示了SMARCA4-DNSCLC恶性程度高、预后差。

综上所述，本研究通过对真实世界的数据分析，发现在SMARCA4-DNSCLC患者中，PD-1单抗联合化疗较化疗疾病缓解率方面无统计学差异，但中位PFS及1年OS率有改善，治疗方案及吸烟史是这些患者PFS的独立影响因素，家族史是OS的独立影响因素。但本研究也存在不足之处：作为单中心回顾性研究，存在信息偏倚可能，且样本量较小，随访时间较短，中位OS数据尚不成熟，PD-1单抗联合化疗组患者中位生存期未到达，仅对1年OS率进行描述。未来仍需大样本、多中心的临床试验来证实我们的结论。同时也需要进一步的基础实验和功能实验进行分子生物学机制的探讨，寻找潜在可能的机制，这也是我们未来研究的一个重要思路和方向。

Competing interests

The authors declare that they have no competing interests.

Author contributions

Wang XJ and Zhang GJ designed the study. Tu M, Jia HX and Liu HP performed the experiments. Wang XJ and Wang Y analyzed the data. Wang YB and Jiang N contributed analysis tools. Lu CY provided critical inputs on design, analysis, and interpretation of the study. All the authors had access to the data. All authors read and approved the final manuscript as submitted.
